# The Cure of Consumption

**Published:** 1880-01-20

**Authors:** 


### *THE CURE OF CONSUMPTION.*

It is now pretty generally believed, universally we might say, in the medical profession, that the age of miracles is over, but the statements now starting the rounds of the medical journals in Germany regarding the cure of tuberculosis by the inhalation of the benzoate of soda are calculated to renew the most sinking faith.

In the number just received of the Wiener Med. Wochenschrift (No. 30, 1879), we read the following notice :

"In the K. K. Allgemeinen Krankenhaus of Vienna, the newly-discovered miracle (wundermittel) of tuberculosis, viz. : the benzoate of soda, has awakened the most intense interest in medical circles everywhere, and inhalations of this substance are now going on in every room of the hospital. Prof. Rokitansky, jr., is credited with the marvelous discovery of the virtues of this age."

The next number (No. 40) contains the following note, addressed to the editor :

"*Greefswald*, *September*, 30*th*, 1879.

"In regard to the newly discovered wundermittel, discovered by Prof. V. Rokitansky, in Innsbruck, for the cure of tuberculosis, the natrum benzoicum, I take the liberty of testifying that I first tried it in tuberculosis processes of the lower animals. My investigations regarding the genesis of scrofulous and tuberculous inflammations of the joints, led me to the conviction that they depended upon a localization of the infecting substance in the artificially contused joints. Definite experiments convinced me that micrococci contained in the tuberculous virus I selected for my inoculations constituted the infecting substance, as was first pointed out by Klebs.

"By a repetition of Klebs' breeding experiments I could collect these micro-organisms, render animals tuberculous by means of them, and thus confirm entirely the statements of Klebs. A part of these my breeding experiments were conducted by one of my former pupils, Dr. Reinstadter, in association with me, and the results were published by him.

"After these demonstrations I commenced some therapeutic experiments, and selected first the benzoate of soda, aqua creosoti, and some other agents, which are known to have an anti-bacterian effect. It now occurred to me to study the effects of these agents on tuberculous affections of the joints. I soon discovered their very remarkable effects and continued my investigations.

"I communicated my first observations to our medical society here, and then published them. Since that time I have been continually at work, and am now able to confirm all the statements made at first.

"It was these observations that induced Prof. V. Rokitansky to try the remedy, the benzoate of soda, on man. I rejoice that he has succeeded in obtaining the same results in man that I obtained in the lower animals, but I may remark that we have already made the same observations upon man here." DR. MAX SCHULLER,

*Privat docent*, *Assistent Arzt der Chirurg. Universitats-Klinik.*

The Wiener Med. Wochenschrift appends to this letter the following remarks :

"This matter is certainly important enough to excite further experiments, even if the cases in which the signs of 'tuberculous cavities' disappeared so rapidly, should turn out to be only bronchiecsatic dilatations after emphysema and chronic catarrh, conditions far more frequently met in Tyrol than pulmonary phthisis, which is rare. For these diseases are also very obstinate to treatment, and are often dangerous.

"We are, moreover, in position to communicate a letter from Dr.

Kroczak, Innsbruck, to one of his patients received a few days ago. The patient had seen in a daily paper an account of the newly dis covered cure of tuberculosis, and addressed himself to the physician, from whom he received the following response :

" 'Our new method of treatment can only be conducted under medical supervision, and may not be properly described by letter. We use one part of benzoate of soda in a 5 per cent. solution twice daily, to the thousand of the body weight, by means of a good atomizer, for seven weeks without interruption. With it we enjoin the use of abundant satisfaction of the rapidly returning appetite with meat diet, fresh air, and abstention from all debilitating causes. DR. KROCZAK.'

"We may add still further," continues the Vienna paper, "that our druggists can hardly supply the demand for the benzoate of soda, as the use of it has surpassed all medical prescriptions. It is, indeed, bought up on every hand."—*J. T. W. in Cin. Lancet and Clinic.*

